# KinaMetrix: a web resource to investigate kinase conformations and inhibitor space

**DOI:** 10.1093/nar/gky916

**Published:** 2018-10-13

**Authors:** Rayees Rahman, Peter Man-Un Ung, Avner Schlessinger

**Affiliations:** Department of Pharmacological Sciences, Icahn school of Medicine at Mount Sinai, New York, NY 10029, USA

## Abstract

Protein kinases are among the most explored protein drug targets. Visualization of kinase conformations is critical for understanding structure–function relationship in this family and for developing chemically unique, conformation-specific small molecule drugs. We have developed *Kinformation*, a random forest classifier that annotates the conformation of over 3500 protein kinase structures in the Protein Data Bank. Kinformation was trained on structural descriptors derived from functionally important motifs to automatically categorize kinases into five major conformations with pharmacological relevance. Here we present KinaMetrix (http://KinaMetrix.com), a web resource enabling researchers to investigate the protein kinase conformational space as well as a subset of kinase inhibitors that exhibit conformational specificity. KinaMetrix allows users to classify uploaded kinase structures, as well as to derive structural descriptors of protein kinases. Uploaded structures can then be compared to atomic structures of other kinases, enabling users to identify kinases that occupy a similar conformational space to their uploaded structure. Finally, KinaMetrix also serves as a repository for both small molecule substructures that are significantly associated with each conformation type, and for homology models of kinases in inactive conformations. We expect KinaMetrix to serve as a resource for researchers studying kinase structural biology or developing conformation-specific kinase inhibitors.

## INTRODUCTION

Protein kinases are a family of signaling molecules that play crucial roles in many disease networks. Changes in protein kinase activity due to mutations or gene expression level often lead to disruptions in their downstream signaling pathways, resulting in maladies such as cancer, neurodegeneration, and autoimmunity (1). As a result, protein kinases constitute one of the largest drug target families in humans (1). Kinases are amenable to inhibition by small molecules that interact with the well-defined adenosine triphosphate (ATP) binding pocket (2). Various approaches from information science and machine learning have already been applied to this field to predict protein–drug interactions (3–5). Furthermore, protein kinases are highly dynamic proteins; their conformations are dictated by the configurations of the αC-helix and the conserved aspartate-phenylalanine-glycine (DFG) motifs that constitute the ATP binding pocket (6). When both motifs are in the ‘in’ conformation: αC-in/DFG-in (CIDI), this represents the ‘active’ state of the kinase. Other alternate configurations of these motifs: αC-in/DFG-out (CIDO), αC-out/DFG-in (CODI), and αC-out/DFG-out (CODO) represent ‘inactive’ states of the kinase. Each distinct conformation presents a unique shape and physicochemical environment of the ATP binding pocket, thus modulating the enzymatic activities of the kinase (7). While consideration of these kinase conformations is critical for the development of targeted kinase inhibitors, manual classification of kinase structures into any of these categories is not fully automated and requires a trained eye. Additionally, while a webserver exists that classifies known kinase structures from the Protein Data Bank (PDB) (8) into related conformations (9), it does not allow user submission of solved atomic structures or computational models of kinases in unseen conformational states.

Recent articles have shown the importance of developing conformation-specific inhibitors of protein kinases. Rukhlenko *et* *al.* demonstrated that utilizing distinct conformation-specific RAF kinase inhibitors can prevent the paradoxical activation of RAF signalling by inhibitors such as vemurafenib (10). Additionally, they showed that the synergistic effect of two distinct conformation-specific kinase inhibitors occurs at lower concentrations compared to a monotherapy approach. On the other hand, Sultan *et al.* utilized extensive molecular dynamics simulations to explore the energy landscape of the BTK and SRC family of kinases in order to understand their thermodynamically stable conformational states and the transitions between the active and inactive states (11,12). Importantly, they identified stable intermediate conformational states within this landscape with potentially druggable pockets, opening the doors for the discovery of chemically distinct scaffolds targeting these conformations.

We have developed Kinformation (7), a random-forest based classifier that grouped 3569 solved protein kinase structures from the PDB into each of the four major kinase conformations; we also identified an additional fifth conformation: ωCD, representing an intermediate state between the four major conformation classes. Kinformation had achieved an out-of-bag error of <10% on a training set of 264 manually annotated kinase structures. Analysis of the small molecules co-crystalized with the kinases revealed key chemical scaffolds that correlated with conformational selectivity. Subsequently, we enumerated a chemical library of over 29 000 chemical fragments from the co-crystalized small molecules. This library was then clustered into over 10 000 representative substructures. Statistical association tests were then performed between each representative substructure against all conformational states, resulting in a library where each substructure was annotated with its association for a specific kinase conformation.

In this article, we present KinaMetrix, a web resource to investigate kinase conformations and the kinase inhibitor chemical space. On KinaMetrix.com, we host Kinformation, enabling researchers to upload and classify the conformation of their kinase structures. KinaMetrix also presents our custom database of annotated conformations for over 3500 kinase structures found in the PDB. Users will also be able to access our library of conformation-specific chemical substructures, which can potentially serve as building blocks for the design of conformation-specific kinase inhibitors. Finally, KinaMetrix will serve as a repository of high-quality homology models of Typical human protein kinases in the CIDO conformation defined in Kinformation, a highly attractive conformation for protein kinase drug discovery studies.

## MATERIALS AND METHODS

### Kinformation

Two hundred and sixty-four unique kinase structures were annotated manually in each conformation type and constituted our training set of kinase conformations (7). These structures were classified into four main conformations: CIDI (110 structures), CIDO (58 structures), CODI (36 structures), and CODO (28 structures). Structures defined as ωCD (34 structures) have distorted αC-helix or DFG motif. Eight geometric descriptors among 28 evaluated descriptors that best described the positions of both the αC-helix and DFG motif were used as features for a random forest classifier (7). Missing data was imputed using the rfImpute method in the randomForest package in the R programming language (13). All non-vector descriptors were then normalized to a scale of (−1, 1). Kinformation was constructed using a two-stage random forest classification: the first stage trains a model on the imputed data with 1000 subtrees to distinguish DFG classes (DFG-in, -out, and -intermediate); the second stage classifies the αC-helix classes (αC-in and -out) with 1000 sub-trees using both the imputed data and the DFG classes. Kinformation was then used to classify the conformation of the remaining kinase structures in the dataset.

### Chemical substructure enrichment

Small molecules interacting with the ATP binding pocket were exhaustively fragmented using the rCDK package (14). The resultant 29 870 fragments, with at least 10 heavy atoms, were clustered into 10 535 representative substructures using the MolBlocks software (15) with a chemical fingerprint cut-off of 0.90. To test for statistical association of a given substructure to a kinase conformation, a one-sided Fisher’s Exact test was used. A Bonferroni-corrected α of 4.7 × 10^−6^ was used to identify conformation-specific substructures.

### Homology modeling of kinase structures

The classified kinase structures are used as input to automatically generate homology models for all Typical human protein kinases in CIDO conformation using our previously reported method, DFGmodel (16).

### Development of Kinametrix.com

The KinaMetrix server was built using the Shiny framework (https://shiny.rstudio.com) within the R programming language. The user interface was designed using the following libraries: ‘shinyWidgets’, ‘DT’, ‘shinycssloaders’, ‘shinythemes’, ‘shinyBS’, and ‘bsplus’. Chemical structure visualization and PDB validation was accomplished using the ‘rCDK’ (14) and ‘bio3D’ (17,18) libraries. All figures were generated using custom R scripts and packages such as ggplot2 and Plotly. The application is deployed using the Heroku platform (https://www.heroku.com).

## RESULTS

### Query of kinase structures database

**Figure 1.**
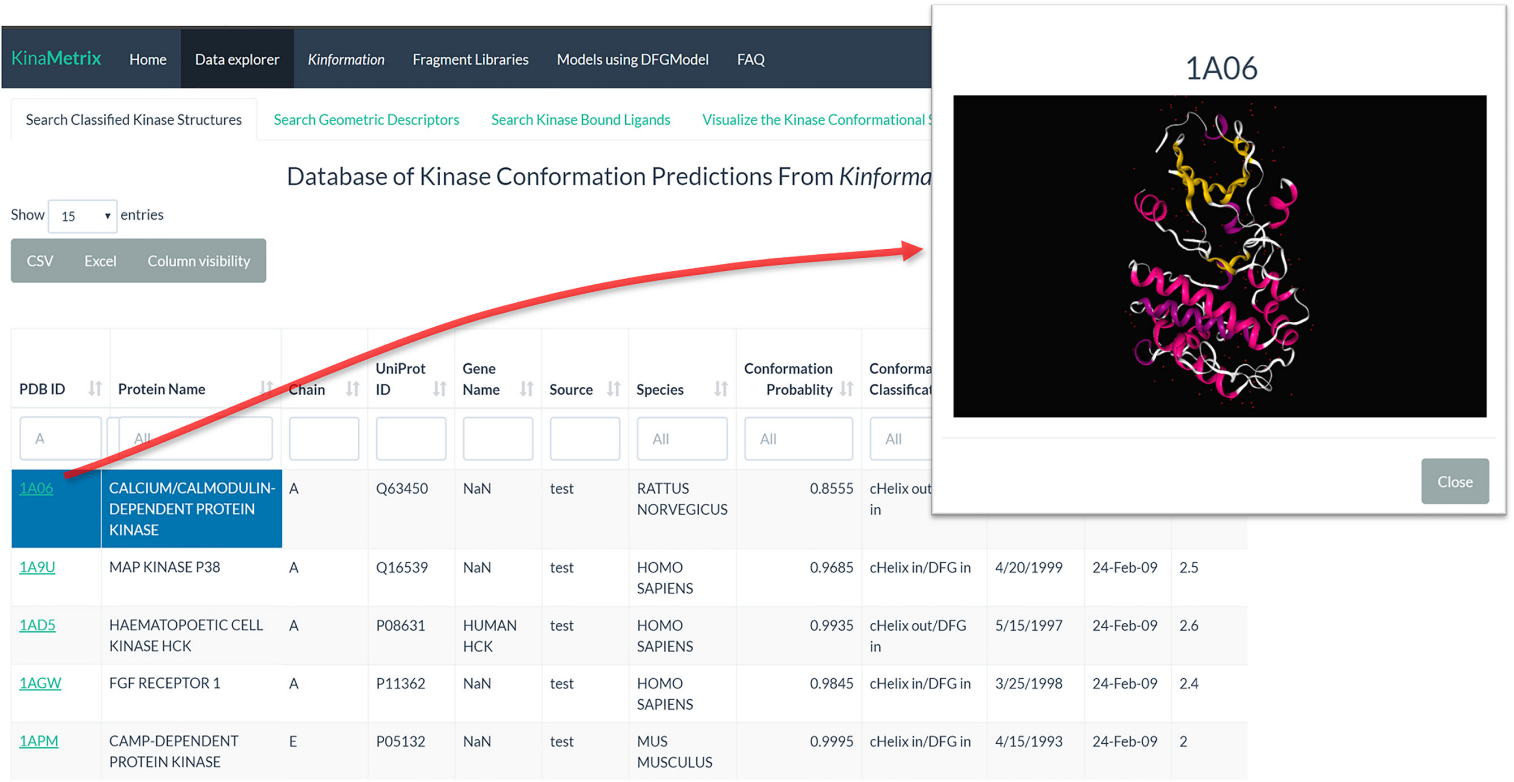
Query classified kinase structures. Under the ‘Data Explorer’ panel, users are able to search through over 3500 conformationally classified kinase structures by their PDB ID, protein name, UniProt ID or gene name. Users can filter the structures by their predicted conformational state. By clicking on an entry’s PDB ID, users can view the structure directly on the site.

Under the ‘Data Explorer’ panel of KinaMetrix.com, users can navigate one of four tabs that searches distinct tables of our custom database of over 3500 kinase structures and query for kinases by their Gene Name, Protein Name, PDB ID or UniProt ID. Under the ‘Search Classified Kinase Structures’ tab, users will be able to obtain the conformation classification as well as the conformation probability for each kinase structure in the database, as determined by Kinformation (Figure 1). Furthermore, users are able to click the PDB ID to directly visualize the kinase structure (Figure 1). Similarly, under the ‘Search Geometric Descriptors’ tab, users can identify the geometric descriptors that describe the conformational space for each kinase structure. This conformational space can be quickly visualized under the ‘Visualize the Kinase Conformational Space’ tab where users can dynamically manipulate a three-dimensional (3D) scatterplot of each kinase structure based on any of the geometric features displayed. Here users can select specific kinases of interest and immediately identify its known conformational landscape; users may also click on any point of this plot and receive a link to the corresponding RSCB PDB webpage for that kinase structure. Finally, users can search for small molecule ligands bound to the kinase structure under the ‘Search Kinase Bound Ligands’ tab by either their PDB ID, or users may search for kinase structures by their PDB ID and discover their bound ligands. From this table, users can visualize each ligand’s chemical structure as well as information regarding their kinase conformational specificity. All tables displayed are available for download as either CSV or Excel files.

### Classification of user uploaded kinase structures

**Figure 2.**
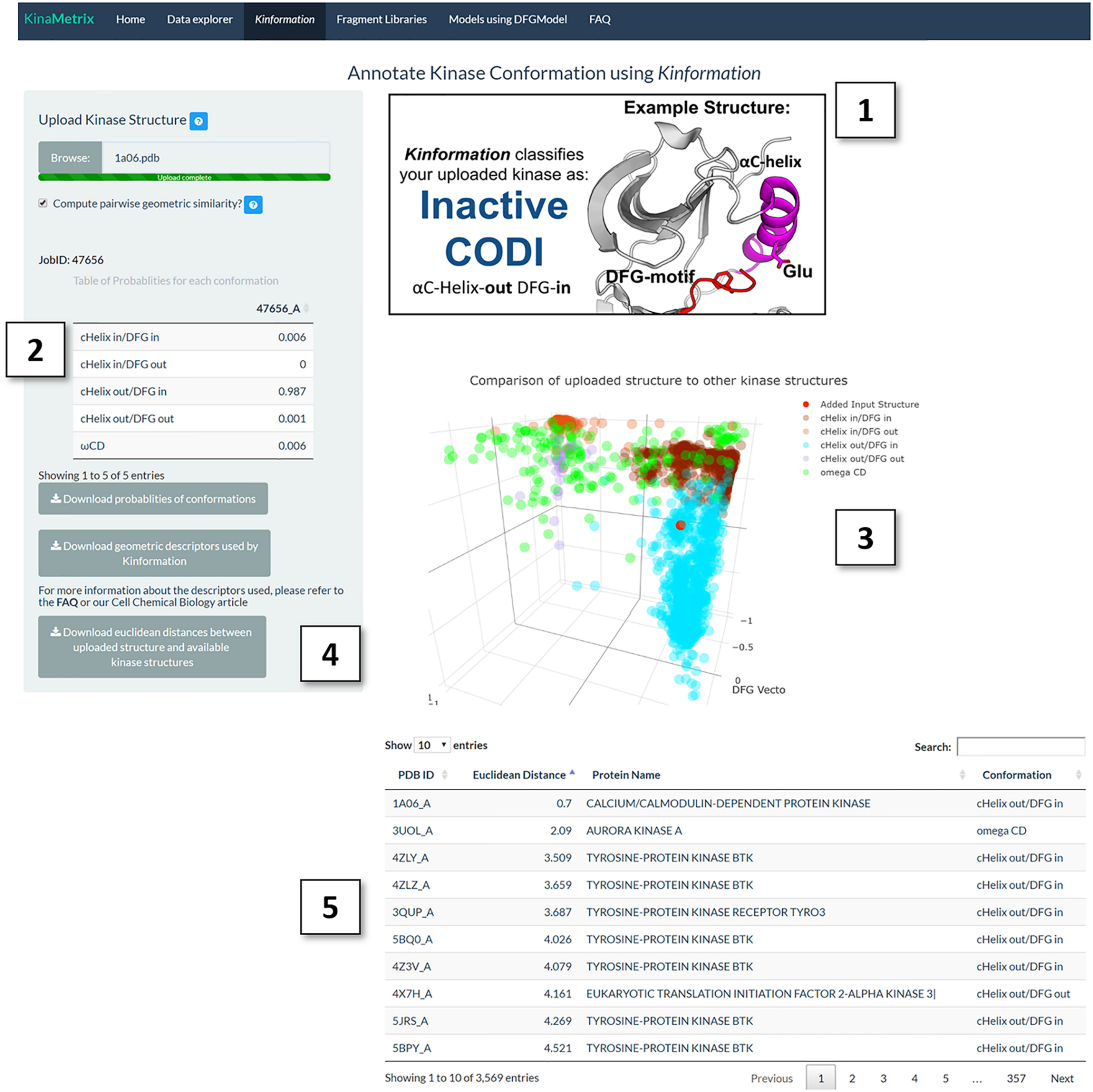
Running *Kinformation* on KinaMetrix. An example output after running *Kinformation* with PDB ID: 1A06 as input. 1A06 corresponds to the structure of Calmodulin-Dependent Protein Kinase. (1) This figure displays the predicted kinase conformation of the user-uploaded structure as well as a prototypical example of the conformation. (2) This table shows the probabilities of all conformational states for the uploaded structure. (3) This plot shows the relative position of the uploaded structure (red dot) in the conformational space described by the αC-helix and DFG motifs in comparison to all other kinase structures available on KinaMetrix. (4) This section allows the user to download the data generated from running the *Kinformation* classifier. (5) This table shows the structural similarity of the uploaded kinase structure to all other kinase structures using the Euclidean distance of their geometric descriptors.

Classification of kinases into the ‘correct’ kinase conformation can help explain the binding mode of known conformation-specific inhibitors or guide the discovery of new inhibitors that target specific conformations. For example, kinases in the CIDO conformation are the most suitable to dock type-II inhibitors such as sorafenib in order to explain their mechanism of action or to develop analogs with unique type-II inhibitor structures (19). Under the ‘Kinformation’ panel, researchers can upload a Typical protein kinase structure in PDB format for classification by Kinformation. KinaMetrix first verifies the file format and then runs Kinformation upon user confirmation (Figure 2). After completion, tables of both the probabilities for each conformation and the computed geometric descriptors are displayed and are available for download as CSV files. The uploaded structure is displayed on an interactive 3D scatterplot with respect to other kinase structures found in the PDB (Figure 2). By checking the ‘Compute pairwise similarity?’ checkbox, users are able to identify other kinase structures with similar geometric descriptors using the Euclidean distance between geometric descriptors of the uploaded structure and all other structures available on the server. As a result, users can identify kinases that occupy a conformational space in the vicinity to that of the uploaded kinase structure; this data can be used in tandem with the ‘Data explorer’ tab allowing users to potentially identify small molecules that stabilize this structural space. All uploaded structures are removed immediately after analysis to maintain confidentiality of the coordinates to their owners.

### Identification of conformation-specific substructures

**Figure 3.**
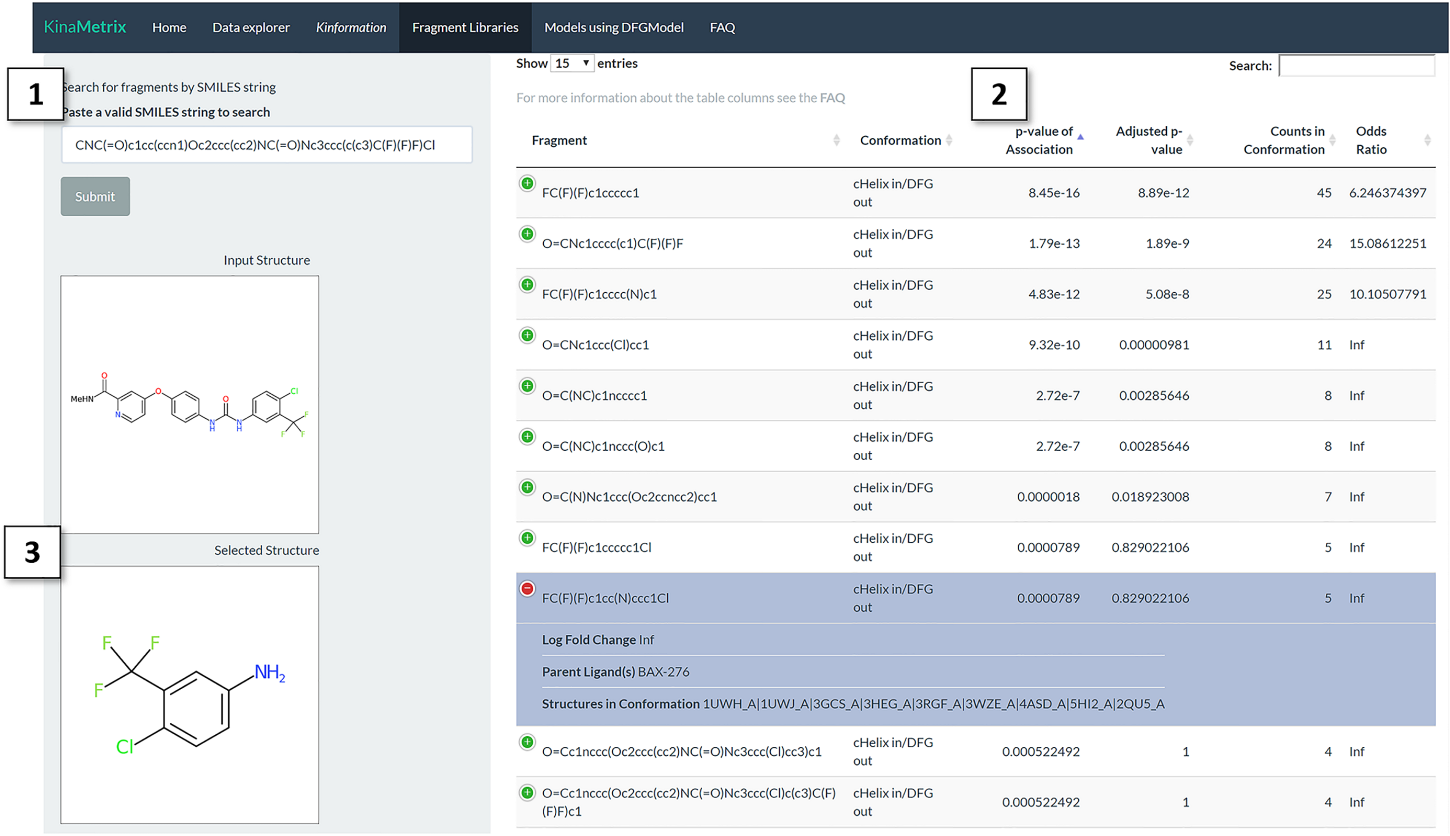
Identifying chemical substructures that confer conformational specificity. Under the ‘Fragment Libraries’ panel and the ‘Search by SMILES’ tab users can search the substructure library on KinaMetrix using a SMILES string. For example, the drug sorafenib was searched against our substructure library. (1) The input SMILES string search box. (2) This table displays the matched substructures to the input chemical. These substructures are annotated with their association for a kinase conformation, as well as information about their parent ligand and their co-crystalized kinase structures. The top substructure matches that are associated with the αC-helix in/DFG out (CIDO) conformational state are shown. (3) The 2D-representation of both the input SMILES string and the selected substructure from the table.

Conformation-specific kinase inhibitors often have specific structural properties that confer their specificity. Therefore, identification of conformation-specific substructures can be used to both classify known small molecules with conformational specificity as well as guide the design of novel conformation-specific drugs. Under the ‘Fragment Libraries’ panel, an interactive Manhattan plot of small molecule substructures enriched for each kinase conformation type is available. Users can highlight, zoom, and select points on the plot to visualize small molecule substructures. In addition to obtaining both the SMILES string and a rendering of the substructure, users will be able to inspect the parent compounds of the substructures, the significance for their kinase conformation association, and investigate the ligand–target interactions through a link to the corresponding RSCB PDB webpage. Under the ‘Search by SMILES’ tab users may also search our substructure library using an input SMILES string, results will show maximum substructure matches for the input SMILES. The resultant matches are annotated with their association for certain kinase conformations and their chemical structure can be visualized alongside of the input SMILES (Figure 3). For example, when searching for the kinase inhibitor sorafenib, all of the top matches described chemical substructures that have high association of CIDO. All information can also be downloaded as a CSV file.

### Access to models of kinases in unseen conformations

The αC-in/DFG-out (CIDO) conformation represents an inactive kinase state that is often selective for type-II kinase inhibitors, an emerging class of drugs with unique pharmacological profiles (1). Structures of CIDO are scarce, limiting the development of novel type-II inhibitors with rational design. We have previously developed DFGmodel, a homology modeling-based method that automatically constructs kinase models in the CIDO conformation, by using a unique set of relevant template structures (9). Under the ‘Models using DFGmodel’ panel users are able to search for and download homology models of Typical human kinases in CIDO conformation generated by DFGmodel. In future updates, we will include homology models of kinases in other major conformations. These models will enable researchers to virtually screen compound libraries to discover conformation-specific tool compounds as well as to inspect physicochemical properties of most kinases in alternative conformations.

### Automated updating of the database based on the availability of new Typical kinase crystal structures

We aim to update KinaMetrix.com periodically as new structures of Typical kinases are solved. The updating of our database is automated using custom R and Python scripts that monitor updates on the RSCB PDB website.

### Future directions

Our goal for KinaMetrix is to develop a repository for data describing the kinase conformational landscape. For this version, we focus on the movements of the αC-helix and DFG motifs. In the future we aim to (i) explore other structural elements, such as the C-Spine and R-Spine (20,21), in order to describe additional conformational spaces applicable for inhibitor development, as well as allosteric pockets integral for other inhibitor types; (ii) to grow our library of conformation-specific substructures by incorporating small molecule imported from ChEMBL (22); and (iii) to continue modeling kinases in uncharacterized conformations using updated versions of our DFGmodel method.

## CONCLUSION

Thorough inspection and investigation of the kinase conformational space will enable new insights into the structural biology of protein kinases as well as guide the development of targeted conformation-specific kinase inhibitors. By creating KinaMetrix.com, we developed a server that enables researchers to easily obtain conformational information of the existing 3500 Typical protein kinase structures deposited in the PDB, classify uploaded kinases structures with high accuracy, obtain high-quality models of Typical kinases in unavailable conformations, and develop novel kinase inhibitors using the knowledge of existing conformation-specific chemical substructures. We expect KinaMetrix.com to serve as a valuable resource for researchers studying protein kinase biology or aiming to develop the next generation of conformation-specific kinase inhibitors.

## DATA AVAILABILITY

All methods are currently available on http://www.KinaMetrix.com. All source code for the web resource can be found on the Schlessinger lab Github repository.
